# 早期肺癌肺叶特异性淋巴结清扫的研究进展

**DOI:** 10.3779/j.issn.1009-3419.2011.01.13

**Published:** 2011-01-20

**Affiliations:** 200030 上海，上海交通大学附属胸科医院/上海市肺部肿瘤临床医学中心胸外科 Shanghai Chest Hospital/Shanghai Lung Tumor Clinical Medical Center, Shanghai 200030, China

**Keywords:** 肺肿瘤, 淋巴结转移, 淋巴结清扫, Lung neoplasms, Lymph node metastasis, Lymphadenectomy

## Abstract

肺癌手术中系统性淋巴结清扫已成为标准术式，但对于早期肺癌尚存在多种清扫方式，各种清扫方式的利弊仍然存在争议。鉴于临床早期肺癌病例日趋增加，特别是Ⅰ期肺癌肺叶特异性淋巴结清扫的研究逐渐深入，本文就目前的相关进展予以综述。

## Abstract (en)

Systematic nodes dissection be the standard procedure for lung cancer during operation. While Surgery for early lung cancer lymph node removal method has been controversial, particularly for clinical early stage non-small cell lung cancer that has presenced several methods of lymph node dissection. In this article, methods of lymph node dissection in the stage Ⅰ non-small cell lung cancer, especially lobe-specifc lymph node dissection of the way, are summarized.

原发性非小细胞支气管肺癌（以下简称肺癌）是目前世界上癌症发病率最高的恶性肿瘤之一，总的5年生存率不足16%^[1]^，临床Ia期肺癌也仅只达到61%-70%^[2, 3]^。外科手术是Ⅰ期、Ⅱ期和部分可切除Ⅲa期肺癌的最佳治疗方式^[4-6]^，但早期尤其是临床Ⅰ期肺癌的淋巴结清扫方式一直存在争议。本文拟就肺癌淋巴结转移的特点和肺叶特异性淋巴结清扫的研究等相关内容进行综述。

## 1 肺癌淋巴结的定位和分区

区域淋巴结状况对于肺癌的临床病理分期、治疗方案的选择评估、患者的预后及治疗效果的评价均具有决定性的作用。为此，Naruke^[7-9]^对肺癌淋巴结进行了详细的分组描绘，并于1978年修订绘制了肺癌N分期的淋巴结分类区域图。Watanabe等^[10]^将纵隔淋巴结区分为上纵隔和下纵隔两个区域，根据肿瘤所在肺叶来确定区域和非区域淋巴结。其中，右侧#1、#2、#3、#4组为上纵隔淋巴结，#7、#8、#9组为下纵隔淋巴结；左侧#1、#2、#3、#4、#5、#6组为上纵隔区域，#7、#8、#9组为下纵隔区域。对于上叶肺癌，上纵隔淋巴结为肿瘤所在区域淋巴结，下纵隔淋巴结则为非区域淋巴结。反之，对于下叶肺癌，下纵隔淋巴结为肿瘤所在区域淋巴结，上纵隔淋巴结则为非区域淋巴结。对于右中叶肺癌，则除上纵隔淋巴结为肿瘤所在区域淋巴结外，尚包括隆凸下淋巴结（#7）。

## 2 肺癌淋巴结的转移方式

肺癌淋巴结转移方式按其淋巴结引流方向存在一定的顺序，即肺内淋巴结→肺门淋巴结→纵隔淋巴结的顺序引流途径发生转移，部分因为引流途径不同而有差异。肺癌的胸内淋巴结引流分为两种模式，即肺的淋巴结引流和纵隔淋巴结引流模式；其中肺的淋巴结引流又分为肺实质内和支气管肺内淋巴结引流，支气管肺内淋巴结引流再分为肺叶和肺门淋巴结引流^[11, 12]^。一般而言，肺癌淋巴结转移常按上述模式依肺内→纵隔顺序发生所在区域淋巴结转移，但亦常出现跳跃式转移和微转移及跳跃式微转移，其淋巴结引流模式和上述模式存在明显不同^[13, 14]^。右上叶肺癌最常发生#3R、#4R组纵隔区域淋巴结转移，其次是上纵隔区域淋巴结转移（#2R)，很少发生下纵隔非区域淋巴结转移；右肺中叶常发生#7纵隔淋巴结转移，其次是上纵隔#2R-#4R组淋巴结转移；右肺下叶常发生#7和下纵隔淋巴结转移，其次亦可以发生上纵隔的非区域淋巴结转移；左上叶最常发生#5、#6组纵隔区域淋巴结转移，其次是上纵隔区域淋巴结转移（#2L -#4L），亦常发生#7组淋巴结转移，较少发生下纵隔非区域淋巴结转移；左肺下叶常发生#7组淋巴结和下纵隔区域淋巴结转移，亦可发生左上纵隔非区域淋巴结转移。此外，有报道左肺下叶肺癌常可发生对侧纵隔淋巴结转移^[10, 11, 15-20]^。跳跃式转移系指肺癌不按肺叶正常的淋巴结引流顺序发生转移，而是越过邻近淋巴结跳跃转移到上一站淋巴结，如跃过N1转移至N2淋巴结^[21]^。有报道^[13]^肺癌的跳跃式转移发生率为20%-38%。肺癌跳跃式转移的机制尚不清楚，可能存在目前未知的肺淋巴结引流模式。研究^[13, 14, 21-25]^认为肺癌跳跃式转移与肿瘤的部位、大小和病理类型有关，如上叶病灶较下叶者易发生，并以左上叶最多见；肺腺癌较常见跳跃式淋巴结转移；#7组淋巴结是最常发生跳跃式转移的淋巴结；发生跳跃式N2转移的患者生存率高于顺序N2转移者；单站跳跃式转移患者生存率高于多站跳跃式转移者。淋巴结微转移是指普通的病理染色（hematoxylin-eosin, HE）无法检测到的肿瘤病灶，其病灶直径常在0.2 mm-2 mm的范围内，更小直径的肿瘤灶则称为孤立肿瘤细胞团，有研究^[26, 27]^认为这种病灶与肿瘤分期及生存率关系不大。微转移被认为是肺癌尤其是早期肺癌转移复发的重要机制之一。HE病理染色阴性的淋巴结中存在微转移肿瘤细胞团可导致肿瘤局部复发转移^[28]^，而跳跃式转移常和微转移相伴发生，两者是从不同角度阐述肺癌淋巴结转移的方式。临床上常将跳跃式转移、微转移、跳跃式微转移称为淋巴结隐匿性转移（occult micrometastases），是早期肺癌最常见的复发转移方式之一。

## 3 肺癌隐匿性淋巴结微转移的检测

对隐匿性淋巴结微转移，HE染色常常将之遗漏，往往需要免疫组织化学（immunohistochemistry, IHC）方法才能够发现微转移病变的淋巴结和单个孤立的癌细胞^[29, 30]^。Passlick等^[31]^认为隐匿性的淋巴结转移是肺癌局部复发的主要原因之一，Ishida等^[32]^也认为淋巴结转移明显增加了原发病灶的扩散。淋巴结微转移的检测手段较多，经典方法是采用针对上皮细胞及其遗传成分的抗体检测淋巴结内是否存在肿瘤细胞，最常用的上皮标志物为细胞角蛋白AE1/AE3，其次为Ber-EP4，这两个抗体既可单独用于检测^[33, 35-38]^，也可联合应用^[34, 39]^，此外，针对遗传基因异常成分检测的一些抗体亦可应用^[40]^，但到目前为止，尚没有一个公认的标准方法。Vollmer等^[33]^应用抗AE1/AE3抗体检测淋巴结微转移，发现该技术检测出的微转移数量较常规HE染色明显增多，可提高17%的患者的病理分期。目前用于检测微转移的方法还有：逆转录聚合酶链反应（reverse transcription-polymerase chain reaction, RT-PCR）^[30, 41-43]^、流式细胞技术^[44]^、细胞印记技术^[45]^等。Hashimoto^[46]^描述了一种新颖的RT-PCR检测方法，应用嵌合式RT-PCR方法针对肿瘤细胞*p53*或者*K-ras*基因突变进行检测，籍以提高检测的敏感性和特异性，可以发现常规病理为pN0患者淋巴结中29%的微转移病灶，此技术的缺点是仍会遗漏无*p53*或*K-ras*基因突变者的微转移病灶。流式细胞技术的优点是可以在短时间内得到结果反馈，研究证实其和IHC有同样的检测微转移效果，但是存在较高的假阳性率，但其对细胞角蛋白AE1/AE3的高速识别却为微转移的检测提供了相对快捷的方法^[44]^。此外，Liptay ^[22]^利用^99^锝硫胶体物标记前哨淋巴结，可以有效地检测出常规病理方法阴性的淋巴结微转移灶。尽管淋巴结微转移的检测手段很多，但许多方法仍处于试验阶段，也没有堪称金标准方法，且由于成本/效益和时效性等多种因素的影响，临床实际应用仍有很大限制。

## 4 肺癌淋巴结的清扫方式

对于肺癌术中淋巴结的清扫方式及范围尚未达成广泛一致，为此，欧洲胸外科医师协会制定了肺癌淋巴结清除方式的定义、手术操作规范及淋巴结病理检查标准指南^[47]^。该指南将淋巴结清扫方式分为5类：（1）选择性淋巴结活检（selected lymph node biopsy），仅对几个可疑淋巴结进行病理检查以确定N分期，主要用于剖胸探查不能手术切除的患者。（2）采样及系统性采样（sampling or systematic sampling），采样指基于手术前影像学或手术中发现，切取几个有代表性的淋巴结；系统性采样指根据原发肿瘤特点切除预先选定的几站区域淋巴结。常用于早期肺癌的手术治疗及手术分期。（3）系统性淋巴结清扫（systematic node dissection, SND），系统性清除解剖标志内包含淋巴结在内的所有纵隔组织，要求最少切除3站纵隔淋巴结，并且其中必须包括隆突下淋巴结，除纵隔淋巴结以外，肺门和肺内淋巴结必须一并切除，被认为是局部肺癌标准术式的重要组成部分。（4）肺叶特异性系统性淋巴结清扫（lobe-specific systematic node dissection），根据原发肿瘤所在肺叶的不同，清除特定区域内包含淋巴结在内的纵隔组织，近年来被一些学者提倡作为早期肺癌手术淋巴结清扫方式的更好的选择。（5）扩大性淋巴结清扫（extended lymph node dissection）：通过胸骨正中切口或颈部切口清除双侧纵隔及颈部淋巴结，曾用于肺癌彻底性根治术中淋巴结清扫的方式而尝试，但其手术并发症及手术风险增加，目前已不再被采纳。

## 5 肺癌不同淋巴结清扫方式的临床价值

传统的观念认为肺癌术中行系统性淋巴结清扫能够得到更好的生存获益，其总的生存率（overall survival, OS）和无病生存率（disease-free survival, DFS）获得提高，同时可以提供更精确的N分期，从而为术后的治疗提供可靠的依据。但也有一种观点认为，对临床Ⅰ期患者术后生存率而言，系统性淋巴结采样/肺叶特异性淋巴结清扫与系统性淋巴结清扫相比没有统计学差异，系统性淋巴结清扫尚可明显提升术后并发症（肺泡及支气管胸膜瘘、喉返神经损伤、食管损伤等）的发生率，并增加术中出（输）血量和术后胸引流量。Keller等^[48]^采用非随机对照方法，比较可手术切除Ⅱ期-Ⅲa期肺癌患者的淋巴结清扫方式，结果显示，采用系统性淋巴结清扫术的患者生存率高于系统性淋巴结采样者。Izbicki等^[49]^报道了169例临床Ⅰ期-Ⅲa期肺癌手术，分别行系统性淋巴结清扫（*n*=76）和系统性淋巴结采样（*n*=93），结果显示两组5年生存率无差别，原因可能是纳入的研究对象中大部分经术后病理证实已经存在纵隔淋巴结转移（N2），其结果还显示，对于肺癌分期，系统性淋巴结清扫比系统性采样更准确。Sugi等^[50]^对115例直径 < 2 cm的周围型肺癌分别行系统性淋巴结清扫和系统性淋巴结采样（*n*: 59 *vs* 56），此项随机研究证实，系统性纵隔淋巴结采样的生存期优于系统性纵隔淋巴结清扫（总体5年生存率：84% *vs* 81%），同时还证实与系统性清扫相关的病死率明显高于系统性采样（23.8% *vs* 3.4%），作者认为对于肿瘤直径 < 2 cm的周围型肺癌可以不需要行彻底的系统性清扫。而国内的前瞻性随机对照研究^[51]^则认为，相对于系统性淋巴结采样，系统性淋巴结清扫明显提高患者者5年生存率。Wright等^[52]^对3个行系统性淋巴结清扫和淋巴结采样的前瞻性研究进行了*meta*分析，结果提示对于行系统性淋巴结清扫的患者术后死亡风险有所减小（HR=0.78, 95%CI: 0.65-0.93, *P*=0.005），然而肺癌术中淋巴结切除是否有生存获益仍然存在争议，其确切性尚不清晰。

## 6 早期肺癌淋巴结清扫方式的抉择

目前所有的研究报告大多总括Ⅰ期-Ⅲa期病例，而对于单纯临床Ⅰ期肺癌，系统性淋巴结清扫是否可使患者得到更好的生存获益，目前还没有大规模的前瞻性随机对照研究。随着肺癌早期诊断技术的进展和理念的更新，临床Ⅰ期病例逐年增多，事实证明多数患者鲜见非区域淋巴结转移，促使胸外科医师反思系统性淋巴结清扫对此类患者的必要性。对于Ⅰ期肺癌淋巴结的清扫方式，目前争议的焦点在于，系统性淋巴结清扫是否有利于患者生存率的改善及分期。Okada等^[53]^将临床Ⅰ期肺癌患者分为两组，一组377例行肺叶特异性淋巴结清扫，另一组358例行系统性淋巴结清扫，其将肺叶特异性淋巴结清扫定义为：如下叶肿瘤未累及肺门及下纵隔淋巴结，则上纵隔淋巴结无需清扫；如上叶肿瘤的肺门及上纵隔淋巴结术中未发现转移，则下纵隔淋巴结无需清扫。该项研究结果显示，在无病生存率和总生存率方面，两种淋巴结清扫方式无明显差异；进一步多因素分析提示肺叶特异性淋巴结清扫方式对无病生存率无明显影响，两组术后相关病死率、肿瘤远处转移和局部复发率无明显差异。作者由此认为，对于临床Ⅰ期肺癌，行肺叶特异性纵隔淋巴结清扫可以达到与系统性淋巴结清扫相同的临床疗效，且前者更符合外科的微创理念。有作者对临床Ia期和病理T1患者的淋巴结清扫方式进行了对比研究，结果显示系统性淋巴结清扫组的术后并发症发生率明显高于系统性淋巴结采样组（26.2 *vs* 11.1%, *P*=0.045），而两组的总生存率和无病生存率无明显差异^[54]^。同时，该研究还发现，肿瘤直径 < 2 cm者和肿瘤直径2 cm-3 cm者的预后存在差异：肿瘤直径 < 2 cm者，两组的5年总生存率和无病生存率相似；肿瘤直径2 cm-3 cm者，系统性淋巴结清扫组的5年总生存率和无病生存率分别为81.6%和77.9%，而系统性淋巴结采样组分别为55.8%和52.5%。作者因此建议，对临床Ⅰ期的肺癌可根据肿瘤大小采用不同的淋巴结清除方式，可由术中病理测量肿瘤直径再决定淋巴结清扫方式。一般认为，系统性淋巴结清扫有利于发现淋巴结微转移灶，从而使术后病理分期更准确，然而术式对肺癌分期的确定性作用并未被广泛接受。曾有两项研究报道系统性淋巴结清扫有利于提高肿瘤分期的准确性，但鉴于一项研究^[55]^中无对照组，而另一研究^[56]^对照组的淋巴结清扫数量极其有限，故结论可容置疑。相反地，一些研究者发现，系统性淋巴结清扫对提高N分期的精确性并无明显优势，Keller及Izbicki等^[48, 57]^发现，无论系统性淋巴结清扫较系统性淋巴结采样所清除的淋巴结数量增加多少，术后两组病理发现的N1或N2病例数百分比相近；此外，多因素分析发现，系统性淋巴结清扫组累及N2的病例数较系统性淋巴结采样组增加。虽然系统性淋巴结清扫术为胸外科医生普遍认可和采用，但迄今尚缺乏大规模前瞻性随机研究的证据显示较其它淋巴结清扫方式有更大生存获益。第90届美国胸外科学会年会（American Association for Thoracic Surgery, AATS）上，Gail等^[58]^公布了一项北美多中心合作的大规模前瞻性随机研究，随访时长6年以上，1, 111例非小细胞肺癌患者，均为病理T1或T2/N0或N1（非#10组淋巴结），所有患者均在术中行淋巴结采样并立即送病理检查，右侧采取#2R、#4R、#7和#10，左侧采取#5、#6、7和#10，上述淋巴结均无转移的患者（共计1 023例）随机分为纵隔淋巴结采样（简称采样）组和纵隔淋巴结清扫（简称清扫）组，其中采样组498例，清扫组525例，术后随访至少5年以上，平均随访6.3年。结果显示：两组的生存率、病死率和死亡率无统计学差异；中位生存期分别为8.1年（采样组）*vs* 8.5年（清扫组）（*P*=0.531）；局部复发转移亦无统计学差异（*P*=0.126）。结论是，系统性纵隔淋巴结清扫对于经术中采样阴性的早期肺癌患者并没有显著的生存率改善，同时也未减少肿瘤的局部和远处复发率。

## 7 结语

综上所述，就临床实际而言，针对早期肺癌，特别是病灶为T1a的病例，肺叶特异性淋巴结清扫是一可选的手术方式。而对于T1b或T2病例，则可考虑先行系统性淋巴结采样，若实时病检皆为阴性，则不必强调系统性淋巴结清扫，反之，若采样标本发现淋巴结转移，仍继以系统性淋巴结清扫为妥。虽然，我们仍期待更具说服力的大规模前瞻性随机对照研究的证据，但从采样到系统采样，进而到系统清扫，再到针对日益增多的早期病例应用肺叶特异性淋巴结清扫或系统性淋巴结采样，也或是一种理性的回归，此非惟科学，尤着眼于患者的福祉，值得关注和探究。
